# A Phase III, Double-Blind, Randomized, Multicenter, Clinical Trial to Evaluate the Efficacy and Safety of a Fixed-Dose Combination of Metformin Hydrochloride and Myo-Inositol Compared to Metformin in Patients With Polycystic Ovary Syndrome

**DOI:** 10.7759/cureus.75616

**Published:** 2024-12-12

**Authors:** Alka Kriplani, Parikshit Tank, Purnima Singh, Neha Maini, Shymala Kaitala, Lakshmi Kantha G, Joydip Paul, Sushma Shah, Urvashi Bhatara, Archana Sinha, Taruna Singh, Shalini Srivastava, Abhijeet Malvi

**Affiliations:** 1 Department of Obstetrics and Gynecology and Assisted Reproductive Technology, Paras Health, Gurugram, IND; 2 Department of Obstetrics and Gynecology and Assisted Reproductive Technology, All India Institute of Medical Sciences, New Delhi, New Delhi, IND; 3 Department of Obstetrics and Gynecology, Ashwini Maternity and Surgical Hospital, Mumbai, IND; 4 Department of Obstetrics and Gynecology, Pushpanjali Hospital, Agra, IND; 5 Department of Obstetrics and Gynecology, Charak Hospital and Research Center, Lucknow, IND; 6 Department of Obstetrics and Gynecology, Andhra Medical College, King George Hospital, Visakhapatnam, IND; 7 Department of Obstetrics and Gynecology, Mysore Medical College and Research Institute, Mysuru, IND; 8 Department of Obstetrics and Gynecology, College of Medicine and Sagore Dutta Hospital, Kolkata, IND; 9 Department of Obstetrics and Gynecology, Sheth V.S. General Hospital and Smt. Nathiba Hargovandas Lakhmichand Municipal Medical College, Ahmedabad, IND; 10 Department of Obstetrics and Gynecology, N.R.R. Hospital, Bangalore, IND; 11 Department of Obstetrics and Gynecology, Indira Gandhi Institute of Medical Sciences, Patna, IND; 12 Department of Obstetrics and Gynecology, Vatsalya Hospital Multispeciality Center, Varanasi, IND; 13 Department of Obstetrics and Gynecology, Om Surgical Center and Maternity Home, Varanasi, IND; 14 Department of Medical Affairs, Eris Lifesciences Ltd., Ahmedabad, IND

**Keywords:** insulin resistance, menstrual irregularity, metformin, myo-inositol, polycystic ovary syndrome (pcos)

## Abstract

Background

Polycystic ovary syndrome (PCOS) poses a significant health concern among reproductive-aged women and is characterized by ovarian dysfunction, hyperandrogenism, and insulin resistance. This study aims to assess the efficacy and safety of metformin and myo-inositol combination therapy compared to metformin monotherapy in patients with PCOS.

Materials and methods

This was a phase III, double-blind, randomized controlled clinical trial. A total of 196 patients with PCOS were randomized in a 1:1 ratio to receive either a fixed-dose combination of metformin hydrochloride 500 mg and myo-inositol 600 mg (Met-Myo) or metformin 500 mg alone (Met) twice daily for 24 weeks. The primary study endpoints were improvement in insulin resistance (homeostatic model assessment of insulin resistance, HOMA-IR) at week 24 and improvement in menstrual cycle disturbances at 12 and 24 weeks.

Results

The Met-Myo combination demonstrated a significantly superior response with 63 (75%) patients showing improvement in HOMA-IR compared to 54 (60.67%) in the Met group (p = 0.049) at week 24. The improvement in the number of patients with heavy menstrual blood flow (>80 mL) was significantly greater in the Met-Myo group (four patients, 4.76%) compared to the Met group (six patients, 6.74%) at week 24 (p = 0.029). Improvement in the percentage of patients with normal menstrual frequency and infrequent menstruation from baseline to week 24 was significantly greater in the Met-Myo group compared to the Met group (p = 0.049). Safety assessments revealed a low and comparable incidence of mild adverse events.

Conclusion

Metformin-myo-inositol combination therapy is superior to metformin monotherapy in addressing menstrual irregularities and improving insulin resistance in PCOS patients, thereby providing a promising avenue for optimizing the management of PCOS.

## Introduction

Polycystic ovary syndrome (PCOS) is one of the most prevalent heterogeneous endocrine disorders affecting women of reproductive age, impacting their quality of life and reproductive health [1]. PCOS is a global health concern with an estimated global prevalence ranging from 8% to 13%, with a significant proportion, up to 70%, remaining undiagnosed [2]. In India, the prevalence of PCOS is wide, ranging from 3.7% to 22.5%, depending on the studied population and diagnostic criteria [3].

The etiology and pathophysiology of PCOS remain intricate, involving a dynamic interplay among genetic, environmental, and hormonal factors, with insulin resistance (IR) playing a significant role [4]. Approximately 50%-70% of women with PCOS have IR, which contributes to hyperandrogenism. This condition is responsible for many PCOS symptoms, including irregular menstrual cycles [5].

Lifestyle modifications, hormonal therapies, and insulin-sensitizing agents constitute the cornerstone of therapeutic interventions [6]. IR, a central pathophysiological factor in PCOS, has prompted the exploration of insulin-sensitizing agents like metformin and myo-inositol as a therapeutic strategy [6,7]. Beyond glycemic control, metformin exhibits favorable effects on ovarian function, ameliorating IR and hyperandrogenism along with improving menstrual regularity and ovulation [5]. Concurrently, myo-inositol has emerged as a promising adjunct in PCOS management. With demonstrated benefits in improving ovarian function, insulin sensitivity, menstrual regularity, and metabolic profiles, myo-inositol complements the therapeutic landscape of PCOS [8-10].

Since the two insulin-sensitizing agents operate through different mechanisms, combining them may yield synergistic effects, enhancing both metabolic and reproductive outcomes concurrently [11]. Considering their individual merits, the combination presents a compelling strategy in PCOS management as evidenced in some studies, yielding enhanced outcomes compared to monotherapies [9,11]. Therefore, this phase III clinical trial was conducted to assess the efficacy and safety of the fixed-dose combination (FDC) of metformin and myo-inositol in patients with PCOS.

## Materials and methods

This phase III, double-blind, randomized, multicenter, prospective clinical trial was conducted at 10 centers across India from 07/04/2021 to 24/02/2023. The research received approval from the relevant Institutional Ethics Committee overseeing the study. The trial adhered to the principles of the Declaration of Helsinki and the Consolidated Standards of Reporting Trials (CONSORT) guidelines. Informed consent was obtained from all participants prior to commencement.

Inclusion and exclusion criteria

Adult females (18-40 years) diagnosed with PCOS based on the Rotterdam criteria constituted the study cohort if they had any two of the three manifestations: hyperandrogenism; disturbed ovulatory function with chronic oligomenorrhea or amenorrhea after negative screening pregnancy test; polycystic ovary as shown by ultrasonography. These patients also had established IR as assessed by the homeostatic model assessment of insulin resistance (HOMA-IR) model, supported by clinical evidence. Pregnant or lactating women, patients with Cushing's syndrome, late onset of congenital adrenal hyperplasia, androgen-secreting tumors, and hormonal dysfunction were excluded from the study.

Study visits

The study encompassed six visits: screening, randomization, and follow-ups at week six, 12, 18, and 24.

Treatment allocation

During screening visits, patients were evaluated for inclusion and exclusion criteria. Randomization occurred at visit 2, allocating all the subjects in a 1:1 ratio by fixed randomization using the randomization sheet for the study to receive either FDC of metformin hydrochloride (HCl) 500 mg (sustained release, SR) plus myo-inositol 600 mg twice daily (Met-Myo group) or metformin HCl (SR) 500 mg twice daily (Met group) for 24 weeks. The sample size was calculated based on the adequate power required to achieve the primary endpoint. At least 173 patients were required, assuming the frequency of improved menstruation in about 30% of patients in the Met group and assumed difference of Met-Myo and Met groups as 20% at 90% power with 5% level of significance.

Laboratory assessments, monitoring, and data collection

**Figure 1:**
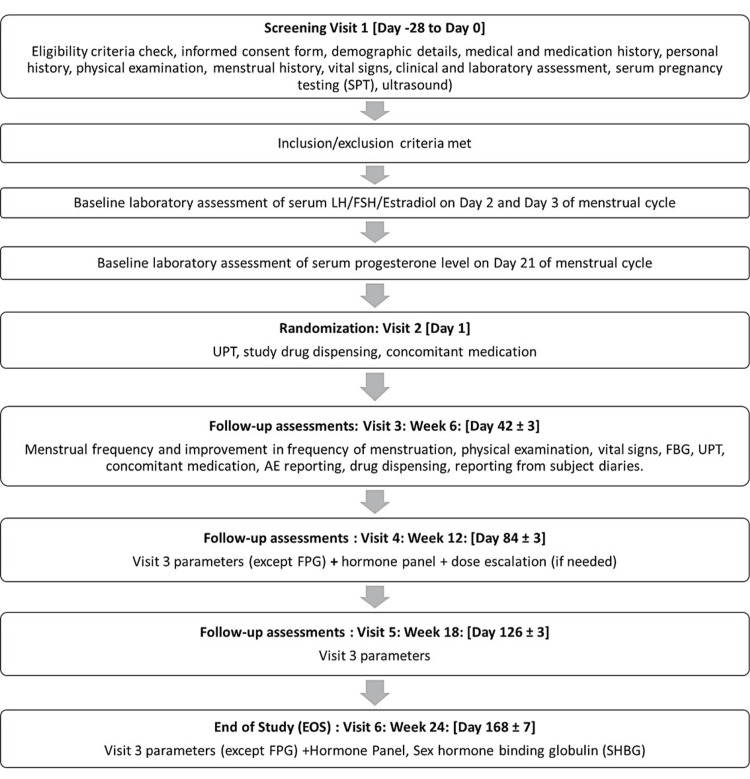
Study flowchart. AE: adverse event; EOS: end of study; FBG: fasting blood glucose; N: number of subjects; UPT: urine pregnancy test; LH: luteinizing hormone; FSH: follicle stimulating hormone.

At baseline, demographic data, menstrual history, etc. were recorded in case report form along with other laboratory assessments, as outlined in Figure 1. At each visit, subjects were carefully monitored for all adverse events (AEs). On visits 4 and 6, laboratory assessments were performed for all the subjects that included hematology, fasting blood glucose (FBG), fasting serum insulin, HOMA-IR, lipid profile (total cholesterol (TC), high-density lipoprotein (HDL), low-density lipoprotein (LDL), triglyceride (TG)), serum testosterone, urine pregnancy test (UPT), follicle-stimulating hormone (FSH), luteinizing hormone (LH), estradiol, and progesterone.

Study endpoints

The primary endpoint was the percentage of subjects with improvement in insulin resistance (HOMA-IR) at 24 weeks of treatment in the Met-Myo group vs. the Met group and improvement in menstrual cycle disturbances at 12 and 24 weeks of treatment. Secondary endpoints of the study were changes in serum testosterone levels, blood pressure, BMI, lipid levels, serum estradiol, progesterone, LH, FSH, and sex hormone-binding globulin (SHBG) levels after 24 weeks of treatment. Safety assessment was done by evaluating treatment-emergent adverse events (TEAEs) after 24 weeks of treatment.

Statistical analysis

**Figure 2:**
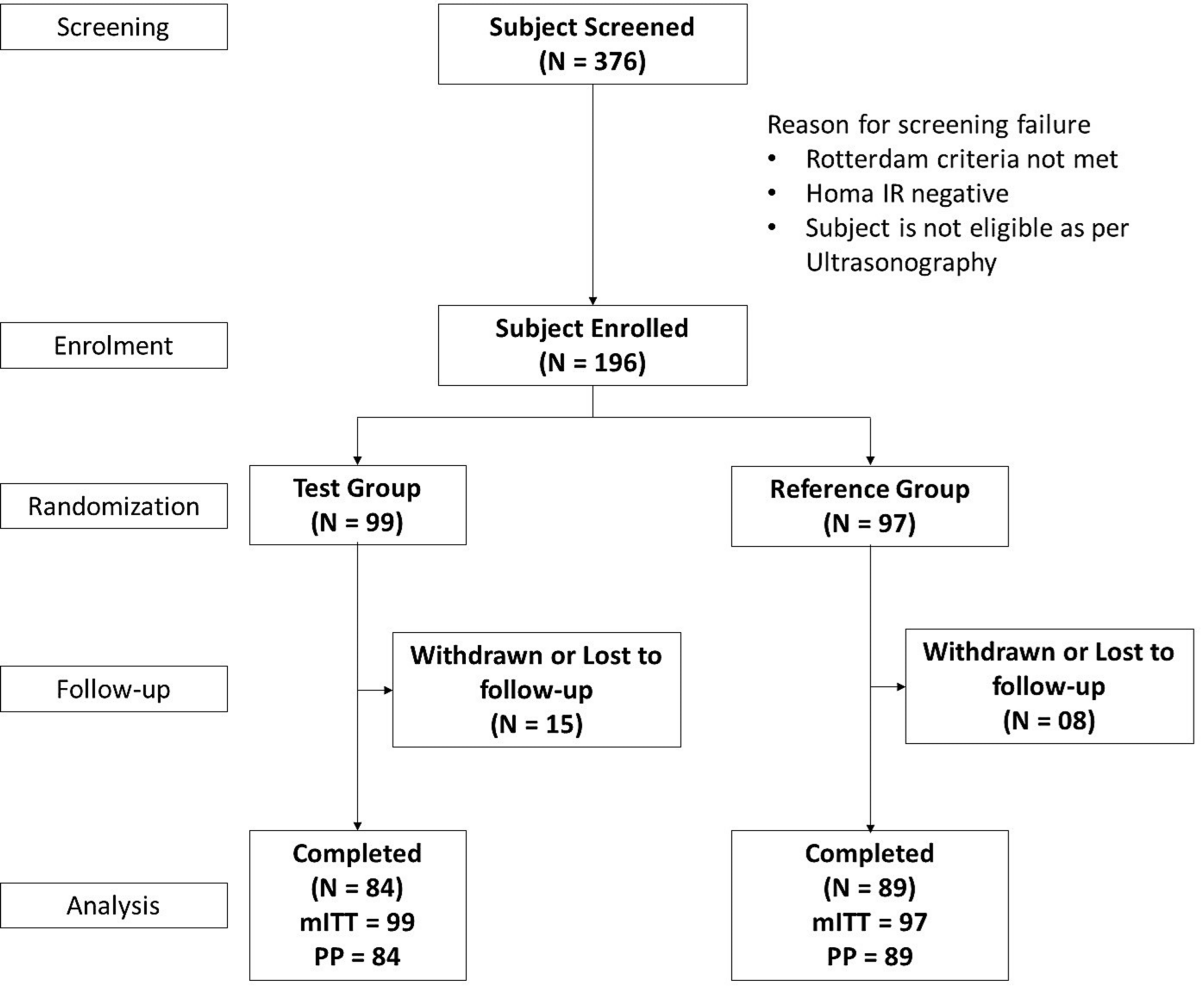
Patient disposition consort flowchart. HOMA IR: homeostatic model assessment of insulin resistance; PP: per-protocol; mITT: modified intent-to-treat.

Appropriate statistical tests were used to analyze data (quantitative data) based on type and distribution (normal and non-normal). In the case of non-normal data, a between-group comparison was performed by the Mann-Whitney test, whereas the data that followed normal distribution were analyzed using the unpaired t-test. For within-group comparison, the paired t-test was used to analyze normal data, and the Wilcoxon test was used to compare non-normal data. In the case of categorical data, data were analyzed using Fisher's exact or chi-square test based on the size of the data. Statistical analysis was performed using GraphPad version 9.4.1 software for Windows (GraphPad Software, San Diego, CA). The statistically significant difference was assumed at p < 0.05. Numerical data were expressed as mean (standard deviation, SD), and categorical data were expressed as the absolute number of subjects. Analysis populations included the modified intent-to-treat (mITT), per-protocol (PP), and safety populations. The efficacy results in the PP population were considered the primary outcome, while the results in the mITT populations were considered supportive. Figure 2 shows the number of subjects who entered this study, were enrolled, and were available for analysis.

## Results

Out of 376 patients who underwent screening, 196 patients were randomized into the study. Throughout the 24-week study duration, 23 patients were discontinued, leaving 173 patients who successfully completed the study. Therefore, the mITT population for statistical analysis comprised all 196 patients, while the PP population for the primary endpoint included 173 patients.

Demographic characteristics

The mean age in the Met-Myo group was 25.38 years, ranging from 18 to 40 years. Comprehensive details of the demographic characteristics are presented in Table 1. Baseline characteristics, including age, BMI, and relevant medical history were comparable between the groups.

**Table 1:** Demographic characteristics of the study population. SD: standard deviation.

Variables, mean (SD)	Met-Myo group (N = 99)	Met group (N = 97)	Overall (N = 196)
Age (years)	25.38 (5.74)	24.70 (5.03)	25.05 (5.39)
Weight (kg)	65.62 (12.49)	65.63 (12.53)	65.63 (12.48)

Efficacy analysis - primary endpoints

Insulin Resistance

Statistical analysis revealed a significantly higher percentage of patients with improved HOMA-IR at week 24 in the Met-Myo group compared to the Met group in the PP and mITT populations (p = 0.049 and p = 0.012, respectively). In the PP population, at week 24, 63 (75%) patients in the Met-Myo group and 54 (60.67%) patients in the Met group showed improved HOMA-IR (Table 2).

**Table 2:** Percentage of patients with improvement in insulin resistance (HOMA-IR) at week 24. * p = 0.049, Met-Myo group vs. Met group (unpaired t-test); ** p = 0.012, Met-Myo group vs. Met group (unpaired t-test); HOMA-IR: homeostatic model assessment of insulin resistance; PP: per protocol; mITT: modified intent-to-treat.

Variables	Met-Myo group (N = 84 in the PP population, N = 99 in the mITT population)		Met group (N = 89 in the PP population, N = 97 in the mITT population)	
Percentage of patients with improved HOMA-IR, n (%)	12 weeks	24 weeks	12 weeks	24 weeks
PP population	56 (66.67)	63 (75.00)*	50 (56.18)	54 (60.67)
mITT population	70 (70.71)	78 (78.79)**	55 (56.70)	60 (61.86)

Absolute HOMA-IR Values

In the PP population and mITT population, the absolute values of HOMA-IR improved from 3.99 ± 2.32 to 2.72 ± 1.47 and 3.90 ± 2.21 to 2.72 ± 1.47, respectively, in the Met-Myo group from baseline to week 24. Similar improvement was seen in the Met group; however, these changes were not statistically significant between the two groups. The overall improvement in HOMA-IR from baseline to week 24 in the Met-Myo vs. Met group was 31.83% vs. 11.05% in the PP population and 32.82% vs. 11.89% in the mITT population.

Improvements in Menstrual Cycle Disturbances

Within the PP population, improvement in the percentage of patients with normal menstrual frequency at baseline to week 24 was significantly more in the Met-Myo group compared to the Met group (p = 0.049) and comparable at week 12 (Table 3). In the mITT population, there was no statistically significant difference between the two groups.

**Table 3:** Improvement in menstrual cycle disturbances at 12 and 24 weeks of treatment in the Met-Myo group vs. the Met group. * p = 0.049, Met-Myo group vs. Met group (unpaired t-test); ** p = 0.029, Met-Myo group vs. Met group (unpaired t-test). PP: per protocol; mITT: modified intent-to-treat.

Variables, n (%)	Met-Myo group (N = 84 in the PP population, N = 99 in the mITT population)			Met group (N = 89 in the PP population, N = 97 in the mITT population)		
	Baseline	Week 12	Week 24	Baseline	Week 12	Week 24
Frequency of menstruation						
Infrequent (>38 days)						
PP population	81 (96.43%)	36 (42.86%)	10 (11.90%)*	82 (92.13%)	37 (41.57%)	19 (21.35%)
mITT population	95 (95.96%)	39 (39.39%)	10 (10.10%)	90 (92.78%)	39 (40.21%)	19 (19.59%)
Normal (24-38 days)						
PP population	3 (3.57%)	47 (55.95%)	74 (88.10%)*	6 (6.74%)	50 (56.18%)	67 (75.28%)
mITT population	4 (4.04%)	50 (50.51%)	73 (73.74%)	6 (6.19%)	51 (52.58%)	67 (69.07%)
Regularity of menstruation						
Variation >20 days						
PP population	59 (70.24%)	31 (36.90%)	26 (30.95%)	58 (65.17%)	39 (43.82%)	29 (32.58%)
mITT population	65 (65.66%)	33 (33.33%)	25 (25.25%)	63 (64.95%)	40 (41.24%)	29 (29.90%)
Variation ± 20 days						
PP population	25 (29.76%)	53 (63.10%)	58 (69.05%)	31 (34.83%)	50 (56.18%)	59 (66.29%)
mITT population	31 (31.31%)	56 (56.57%)	58 (58.59%)	34 (35.05%)	52 (53.61%)	59 (60.82%)
Heaviness of menstruation flow (volume of blood loss in mL)						
Heavy (>80 mL)						
PP population	10 (11.90%)	8 (9.52%)	4 (4.76%)**	11 (12.36%)	6 (6.74%)	6 (6.74%)
mITT population	13 (13.13%)	6 (6.06%)	4 (4.04%)**	11 (11.34%)	7 (7.22%)	6 (6.19%)
Light (<5 mL)						
PP population	26 (30.95%)	21 (25.00%)	4 (4.76%)**	25 (28.09%)	23 (25.84%)	15 (16.85%)
mITT population	29 (29.29%)	23 (23.23%)	4 (4.04%)**	27 (27.84%)	24 (24.74%)	15 (15.46%)
Normal (5-80 mL)						
PP population	48 (57.14%)	55 (65.48%)	6 (90.48%)**	53 (59.55%)	60 (67.42%)	68 (76.40%)
mITT population	56 (56.57%)	59 (59.60%)	76 (76.77%)**	59 (60.82%)	61 (62.89%)	68 (70.10%)
Duration of menstrual flow						
Normal (4.5-8 days)						
PP population	46 (54.76%)	52 (61.90%)	72 (85.71%)	42 (47.19%)	48 (53.93%)	63 (70.79%)
mITT population	54 (54.55%)	55 (55.56%)	72 (72.73)	45 (46.39%)	49 (50.52%)	63 (64.95)
Shortened (<4.5 days)						
PP population	34 (40.48%)	28 (33.33%)	11 (13.10%)	42 (47.19%)	38 (42.70%)	24 (26.97%)
mITT population	40 (40.40%)	30 (30.30%)	11 (11.11%)	46 (47.42%)	39 (40.21%)	24 (24.74%)
Prolonged (>8.0 days)						
PP population	4 (4.76%)	4 (4.76%)	1 (1.19%)	5 (5.62%)	3 (3.37%)	2 (2.25%)
mITT population	5 (5.05%)	4 (4.04%)	1 (1.01%)	6 (6.19%)	4 (4.12%)	2 (2.06%)

Similarly, improvement in the number of patients with infrequent (>38 days) menstruation at baseline to week 24 was significantly more in the Met-Myo group compared to the Met group (p = 0.049) in the PP and mITT population and comparable at week 12. There was no statistically significant difference between the two groups in the reduction in the number of patients with variation >20 days from baseline to weeks 12 and 24 in both the PP and mITT populations. The improvement in the number of patients with heavy menstrual blood flow and normal blood flow at baseline to week 24 was significantly greater in the Met-Myo group compared to the Met group (p = 0.029) and comparable at week 12 in both PP and mITT populations.

At baseline, normal blood flow duration was observed in 46 (54.76%) patients in the Met-Myo group and 42 (47.19%) in the Met group. By week 24, this increased to 72 (85.71%) and 63 (70.79%) patients, respectively, with no significant difference between the groups. At baseline, in the PP population, none of the patients in the Met-Myo group and one patient in the Met group had regular menses, and most patients had oligomenorrhea, with 81 (96.43%) patients in the Met-Myo group vs. 84 (94.38%) patients in the Met group, and few having amenorrhea, with three (3.57%) patients in the Met-Myo group vs. four (4.49%) patients in the Met group. By week 12 and 24, both groups showed improvement, but with no significant difference between them. Similar results were observed in the mITT population. None of the patients in the PP and mITT populations had amenorrhea at the end of 24 weeks.

Efficacy analysis - secondary endpoints

Changes in Serum Testosterone Levels

In the PP and mITT population, both the Met-Myo and Met groups showed significant reductions in serum testosterone from baseline to weeks 12 and 24 (p < 0.05), with no significant difference between them (Table 4).

**Table 4:** Changes in laboratory parameters at 12 and 24 weeks of treatment in the Met-Myo group vs. the Met group. * p < 0.05 vs. baseline (within-group comparison, paired t-test); ** p < 0.05, Met-Myo group vs. Met group (unpaired t-test). NA: not assessed; TC: total cholesterol; HDL: high-density lipoprotein; LDL: low-density lipoprotein; TG: triglyceride; LH: luteinizing hormone; FSH: follicle-stimulating hormone; SHBG: sex hormone-binding globulin; PP: per protocol; mITT: modified intent-to-treat.

Variables, mean (SD)	Met-Myo group (N = 84 in the PP population, N = 99 in the mITT population)			Met group (N = 89 in the PP population, N = 97 in the mITT population)		
Visits	Baseline	12 weeks	24 weeks	Baseline	12 weeks	24 weeks
Serum testosterone (mg/dL)						
PP population	46.71 (31.46)	39.44 (24.26)*	38.01 (26.89)*	47.24 (25.74)	43.35 (34.68)*	40.17 (25.93)*
mITT population	47.61 (30.30)	39.57 (25.03)*	38.01 (26.89)*	48.23 (28.85)	43.91 (34.83)*	40.17 (25.93)*
TC (mg/dL)						
PP population	165.5 (34.45)	162.6 (35.51)	156.4 (27.61)	163.9 (31.8)	158.4 (28.57)	164.8 (32.56)
mITT population	166.44 (33.62)	162.2 (35.29)	156.4 (27.61)*	162.65 (32.67)	159.5 (29.82)	164.4 (32.58)
HDL (mg/dL)						
PP population	43.2 (8.72)	46 (10)	46 (9.3)*	44.5 (7.88)	48 (14)	48 (9.2)*
mITT population	43.3 (8.97)	46 (11)	46 (9.3)	44 (8.02)	48 (14)	48 (9.2)*
LDL (mg/dL)						
PP population	101.4 (26.21)	101.9 (27.32)	97.58 (24.57)**	96.37 (27.26)	95.16 (23.12)	104 (23.09)*
mITT population	101.5 (26.24)	101.2 (27.15)	97.58 (24.57)**	95.71 (28.16)	96.1 (24.18)	103.5 (23.49)*
TG (mg/dL)						
PP population	121.7 (53.84)	117 (49.1)	114 (49.3)	118.4 (76.36)	121 (49.2)	115 (47.8)
mITT population	121.5 (54.12)	118 (48.1)	114 (49.3)	117.7 (74.22)	120 (50.9)	116 (47.6)
Estradiol (pg/mL)						
PP population	45.92 (16.80)	85.32 (89.55)*	86.11 (74.04)*	47.02 (25.71)	71.31 (51.09)*	83.14 (64.28)*
mITT population	49.24 (22.84)	84.60 (87.90)*	86.11 (74.04)*	47.42 (25.53)	69.86 (50.89)*	83.14 (64.28)
Progesterone (ng/mL)						
PP population	3.04 (4.63)	2.19 (4.56)	2.04 (4.17)	3.26 (4.99)	1.86 (3.05)*	2.46 (4.37)
mITT population	3.12 (4.90)	2.21 (4.49)	2.04 (4.17)	3.30 (5.47)	1.83 (3.00)*	2.46 (4.37)
LH (mIU/mL)						
PP population	8.55 (6.25)	8.21 (5.31)	6.95 (4.04)	8.05 (5.72)	7.33 (4.40)	7.07 (4.19)
mITT population	8.27 (6.07)	8.24 (5.20)	6.97 (4.02)	7.57 (5.32)	10.44 (7.90)	7.07 (4.19)
FSH (mIU/mL)						
PP population	5.74 (1.80)	5.06 (1.63)	5.31 (2.17)	5.73 (2.02)	5.26 (1.85)	5.23 (2.27)
mITT population	5.66 (1.77)	4.99 (1.68)*	5.31 (2.17)	5.77 (1.98)	5.31 (1.86)	5.23 (2.27)
SHBG (nmol/L)						
PP population	49.01 (33.18)	NA	54.59 (31.00)	51.75 (39.30)	NA	51.47 (33.03)
mITT population	47.57 (31.54)	NA	54.59 (31.00)	50.99 (38.12)	NA	51.47 (33.03)

Changes in Blood Pressure and Hip-Waist Ratio

There were no significant changes noted within the group from baseline to weeks 12 and 24 and also between the treatment arms in the PP and mITT populations.

Changes in BMI

In the PP population, there was a significant reduction in BMI from baseline to week 24 in the Met-Myo (-1.02 ± 1.37) and Met groups (-0.70 ± 1.44, p < 0.05) but not in the mITT population. There was no significant difference between the two groups for both PP and mITT populations.

Changes in Lipid Levels

In the PP population, in both groups, there was no significant change in TC and TG levels at week 24, and HDL cholesterol increased significantly (p < 0.05), while LDL cholesterol increased significantly in the Met group (p < 0.05) and decreased non-significantly in the Met-Myo group. Except for LDL, other lipid parameters showed no significant difference between the groups (Table 4).

Changes in Estradiol, Progesterone, LH, FSH, and SHBG After 24 Weeks of Treatment

There were no significant changes in serum LH, progesterone, FSH, and SHBG levels at week 24 compared to baseline in both PP and mITT populations of both groups. However, there was a significant increase in serum estradiol levels in both groups (p < 0.05). No significant differences were found between the groups for these parameters.

Safety analysis

Safety evaluation was performed for all patients who received at least a single dose of the treatment in either group. Out of 11 TEAEs reported in 10 patients, six TEAEs were reported in the Met-Myo group by five patients while five TEAEs were reported by five patients in the Met group. All the TEAEs were mild in severity and unlikely related to study medication. The most common adverse events recorded were bloating (n = 2), headache (n = 3), fever (n = 1), and nausea (n = 1). None of the patients were discontinued due to AE and TEAE. No serious adverse events or deaths were reported during the study period.

## Discussion

PCOS is the most common endocrine disorder affecting reproductive-age women and the leading cause of infertility among endocrinological disorders. Insulin-sensitizing agents, metformin and myo-inositol, are the first-line pharmacological treatment to restore ovulation and normal menstrual cycles in women with PCOS [12,13]. Several studies explain the better effects of the combined use of metformin and myo-inositol on endocrine, metabolic, and reproductive outcomes [9,11,14]. In our study, the Met-Myo combination emerged as a revolutionary treatment for PCOS management, demonstrating superior efficacy in restoring menstrual regularity and addressing IR compared to metformin alone.

With respect to the primary outcome, our study demonstrated a significant improvement in the percentage of patients with improved HOMA-IR in both groups. The Met-Myo combination showed superior results, with 75% of patients showing improvement compared to 60.67% in the metformin-alone group. These results align with the existing body of evidence supporting the combination’s role in ameliorating IR in PCOS [9,15,16].

Myo-inositol is a second messenger in the FSH signaling pathway and its deficiency is related to ovulatory dysfunction in PCOS [17]. A positive effect of myo-inositol on improving IR, TG, and testosterone was reported in studies done in patients with PCOS [18,19]. A study by Nagaria et al. of PCOS patients treated with metformin and myo-inositol combination showed significant improvement (p = 0.0076) in insulin sensitivity, consistent with our findings [9]. Due to the distinct mechanisms of action exhibited by myo-inositol and metformin in enhancing insulin sensitivity and regulating hyperinsulinemia, the combined use results in an additive effect for improving clinical outcomes [9].

The Met-Myo group showed significant improvements in menstrual frequency and heaviness compared to the Met group over 24 weeks, providing strong evidence that combination is more effective than metformin alone in normalizing menstrual parameters. In a study by Thakur et al., a combination group demonstrated a greater improvement in menstrual irregularities, insulin sensitivity, and HOMA-IR compared to those treated with metformin or myo-inositol alone [20]. In a study by Nagaria et al., a combination of metformin and myo-inositol resulted in a 100% spontaneous resumption of menses in cases of amenorrhea, with 90.09% of cases with oligomenorrhea achieving regular menstrual cycles [9].

In a study by Agrawal et al., the combination group exhibited a significant improvement in HOMA-IR and menstrual cycles, compared to the metformin-alone group (p < 0.05) [11]. In a study by Nazirudeen et al., a notably greater improvement in menstrual cycle regularity was observed with combination therapy compared to metformin alone (p < 0.001) [21].

As evidenced in our study, both treatments contribute to a substantial reduction in testosterone levels, emphasizing their individual efficacy in managing hyperandrogenism. In a study by Nagaria et al., significant improvement in serum testosterone levels was observed (p = 0.0002) with combination therapy [9]. Our study outcome also suggests a potential protective effect of combination therapy in improving HDL cholesterol levels and preventing the adverse increase in LDL cholesterol compared to metformin monotherapy.

Also, the significant increase in estradiol levels with both treatments suggests a positive impact on estrogen production, which may contribute to improvements in menstrual cycle regularity and reproductive outcomes. The absence of severe or life-threatening AEs underscores the safety of Met-Myo combination therapy in the studied population. Importantly, none of the reported AEs led to the discontinuation of treatment in either group.

Study limitations

Despite the promising findings, our study has some limitations that should be considered. Firstly, the relatively short duration of the study (24 weeks) may not capture long-term outcomes and sustained efficacy over an extended treatment period. Secondly, our study focused on specific outcome measures, and the broader impact on quality of life and patient-reported outcomes was not comprehensively evaluated.

## Conclusions

The combination therapy of metformin and myo-inositol is superior to metformin monotherapy in addressing menstrual irregularities, particularly the frequency and heaviness of menstruation, and improving IR in patients with PCOS. Both interventions demonstrated a favorable safety profile and no severe adverse events were reported. These findings highlight the potential of the Met-Myo combination as a superior therapeutic approach for managing PCOS.
